# Quantifying coordinative patterns in steady‐state running: The impact of footwear and foot strike on joint coupling variability

**DOI:** 10.1002/ejsc.12056

**Published:** 2024-03-18

**Authors:** Alessandro Garofolini, Karen J. Mickle, Patrick McLaughlin, Simon Taylor

**Affiliations:** ^1^ Institute for Health and Sport (IHES) Victoria University Melbourne New South Wales Australia; ^2^ School of Environmental and Life Sciences The University of Newcastle Newcastle New South Wales Australia

**Keywords:** biomechanics, footwear, intralimb coordination, joint couplings, running

## Abstract

In this study, we aimed to compare and contrast the intralimb coordinative patterns of habitual forefoot strikers (FFS) and rearfoot strikers (RFS) during steady‐state running across three different shoe types: minimalist, neutral and cushioned shoes. To describe these coordinative patterns, we implemented the concept of the ‘preferred movement path’ which represents the movement path that runners naturally select in response to their physical capacity and external environment. We quantified cycle‐to‐cycle consistency (ACC) and within‐trial variance in coordination patterns (SoV) using joint angle data from the ankle and knee for ankle‐knee coupling and from the knee and hip for knee‐hip coupling. Additionally, we calculated the measure of shape difference in joint coupling as the sum of squared distances (SSD) between/within conditions and groups. The percentage of runners who displayed shape differences below certain SSD thresholds was also evaluated. Our findings revealed no significant group or shoe type effect on any of the variability measures (ACC: *p* = 0.460 for ankle‐knee and *p* = 0.832 for knee‐hip; SoV: *p* = 0.345 for ankle‐knee and *p* = 0.755 for knee‐hip). However, there was a significant (*p* < 0.001) shape difference observed between the most extreme shoe conditions. Despite this, when runners switched shoes, 70%–90% of them maintained their original coordinative pattern across both joint couplings, indicating a strong adherence to their preferred movement path. This suggests that while shoe type can influence the shape of the coordinative pattern, the inherent movement tendencies of the runners remain largely consistent.

## Highlights

- The study found no differences in the cyclogram variability between habitual foot strike classification groups when wearing different shoes.
- The largest number of different movement responses was present at the ankle‐knee coupling.
- A significantly greater proportion of participants changed their ankle‐knee and knee‐hip coordinative patterns between the High versus Low Minimal Index (MI) conditions compared to the other shoe comparisons.
- No differences were found between the groups in terms of cyclogram variability.

## 1 INTRODUCTION

The human locomotor system generates diverse kinematic gait patterns in the lower limb with intralimb coordination tending to simplify into a few motor synergies or modular properties (Ivanenko et al., 2007; Lacquaniti et al., 2012). Addressing natural variability in human movement is crucial for several reasons. First, understanding the range of natural variability can help identify when a movement pattern deviates significantly from the norm, which may be indicative of an impending injury or a pre‐existing condition (Stergiou et al., 2006). Second, variability in movement paths can also be a protective mechanism as it distributes mechanical stress across different joint structures, thereby reducing the risk of overuse injuries (Emken et al., 2007). Lastly, understanding this variability can inform the design of more effective injury prevention programs by targeting the specific aspects of joint coordination and coupling that are most susceptible to injury (Santos et al., 2020). Therefore, studying the natural variability in human movement paths is not only significant for understanding human locomotion but also vital for injury prevention related to joint coordination and coupling alterations.

A thorough investigation into the consistency of intersegmental covariance in running gait is essential for uncovering the preferred movement path of the neuro‐musculoskeletal system (Nigg, 2001; Nigg et al., 2015; Weir et al., 2018). The preferred movement path is quantified using kinematic gait trajectories, while deviations from this path are considered departures from inherent behavior (Nigg et al., 2017). However, it is worth noting that the preferred movement path paradigm may not fully capture the natural variability inherent in human movement, a factor that could be crucial for a more comprehensive understanding of locomotion. Evidence suggests that a certain degree of variation can actually enhance system complexity without requiring active intervention from the nervous system (Scholz & Schöner, 1999; Todorov & Jordan, 2002). Accordingly, researchers propose that trajectories should be considered not solely based on mean values but also take into account similar paths that still achieve desired motor outcomes (Federolf et al., 2018). This approach allows for greater availability of redundant solutions and enhances adaptability within steady‐state activities by broadening movement variability around coordinative patterns while maintaining stable performance levels (Guo & Raymond, 2010; Pekny et al., 2015; Wu et al., 2014).

Previous studies on preferred movement paths only examined individual joint angles (Nigg et al., 2017; Stacoff et al., 2000) without considering the interdependency between joints due to mechanical and neural limitations. Changes in a single joint angle can affect neighboring joints and alter their coupling (Federolf et al., 2013). This change could result from differences in foot strike pattern (Pohl & Buckley, 2008) or shoe features (DeLeo et al., 2004) leading to an abrupt shift of stress on unadapted tissues that may cause overuse injuries. Due to the frequency of these types of injuries, interventions aimed at modifying running mechanics have become increasingly important (Cheung & Davis, 2011; Crowell & Davis, 2011; Davis et al., 2017; Samaan et al., 2014). Recent research has shown that high‐volume runners exhibit different gait mechanics compared with low‐volume runners (Boyer et al., 2014) suggesting training may impact coordinative patterns. It is important to note that injured runners display altered shank‐rearfoot (Rodrigues et al., 2013) and thigh‐shank coordination (Hamill et al., 1999). While these findings highlight the changes in coordination post‐injury, it does not necessarily imply that these altered patterns lead to injuries. Instead, understanding these interdependencies is crucial for injury prevention as they provide insights into potential biomechanical risk factors.

A thorough analysis of intralimb coordination necessitates a precise measurement of the trajectory shape in angle‐angle plots also known as cyclograms. While conventional linear analyses have been commonly used to assess running performance (Hall et al., 2013; Moore, 2016; Williams & Cavanagh, 1987), they fall short in providing insights into the control system that governs coordinated movement (Cavanagh & Grieve, 1973). In contrast, studying cyclograms allows for an exploration of geometric properties (Hershler & Milner, 1980) and offers a comprehensive understanding of how limb segments function together during motion (Bartlett, 2007). The choice of cyclograms as an analytical tool is justified by their established utility in providing comprehensive insights into movement patterns (Porta et al., 2021) and their proven applicability in various biomechanical and rehabilitation contexts (Field‐Fote & Tepavac, 2002). Furthermore, given that different strike patterns–rearfoot or forefoot–require unique kinematic adaptations and temporal adjustments during running movements (Lieberman et al., 2010), it is reasonable to hypothesise that these two styles may exhibit differential intralimb coordinative patterns worthy of investigation.

The aim of the present study was to examine and expand on the intralimb coordinative pattern, as well as its variability among habitual forefoot strikers and rearfoot strikers who were running in various shoe types. Based on previous research, it is possible that runners who habitually use a forefoot strike have developed a more complex system (Garofolini et al., 2022) with greater available degrees of freedom (Paquette et al., 2013; Squadrone & Gallozzi, 2009), which could result in lower cycle‐to‐cycle consistency indices and higher path variability compared to those using a rearfoot strike. Given these differences in foot strike patterns between groups, we hypothesised that there would be distinct mean coordinative patterns for each group particularly at the ankle‐knee coupling compared to the knee‐hip coupling. Furthermore, because gait mechanics (Soares et al., 2018) and running patterns (Chambon et al., 2014; Nigg et al., 2017) are influenced by footwear type or substrate used during running activities, we postulated that minimalist supportive shoes may elicit increased intralimb coordination variability, while highly supportive shoes might have an ‘equalisation’ effect between groups.

## 2 METHODS

### 2.1 Participants

Forty male long‐distance runners volunteered to take part in this study. Participants were recruited using convenience sampling and were included if they had been running for at least 5 years with an average of at least 40 km/week and had been free of neurological, cardiovascular and musculoskeletal injuries within the previous 6 months. Of the 40 volunteers, 21 runners were eligible to participate and provided informed consent prior to data collection. One was unable to complete the study, resulting in 20 participants (age: 31.2 ± 6.9 years, height: 1.77 ± 0.07 m, weight: 73.4 ± 7.9 kg, training load: 83 ± 22.5 km/week and age‐graded score: 67.8 ± 6.4%) completing all data collection sessions. The age‐graded score was computed via (www.howardgrubb.co.uk/athletics/wmalookup06.html) according to runners age, gender and self‐reported best race performance, similar to Liu et al. (2020). All participants were classified as competitive runners given an age‐graded score of >60% (Clermont et al., 2019). Participants were classified as rearfoot strikers (RFS, *n* = 10) or forefoot strikers (FFS, *n* = 10) based on their habitual foot strike tested on an instrumented treadmill (AMTI Pty, Watertown, MA, USA) at their preferred running speed for 5 min wearing their habitual running shoes (more details in Supplementary Table S1). The preferred running speed did not differ between groups (RFS = 12 ± 1.1 km/h; FFS = 12 ± 1 km/h and *p* = 0.9182). The Victoria University Research Ethics Committee has approved the study (No. HRE16‐061).

Habitual foot strike classification was based on data collected in the last minute of running with their own running shoes by computing the ankle joint moment from foot contact to the time of reaching 1 body weight on the vertical component of the ground reaction force. Runners who displayed a positive (dorsiflexor) moment for at least 90% of the analyzed period were classified as rearfoot strikers (RFS); conversely, runners who displayed a negative (plantarflexor) moment for at least 90% of the analyzed period were classified as forefoot strikers (FFS). This classification has been proposed to be more closely aligned with the ankle function compared to conventional methods (Garofolini et al., 2017).

### 2.2 Experimental protocol

After a standardised 7‐min progressive warm‐up, participants ran for five minutes in three different types of footwear with a distinct minimalist index (MI) (Esculier et al., 2015); low MI shoes (Mizuno® Wave Rider 21, MI = 18%); medium MI shoes (Mizuno® Wave Sonic, MI = 56%) and high MI shoes (Vibram® Five fingers, MI = 96%) quantified by the researcher AG for details see Garofolini et al. (2022). Kinematics of the lower extremities were evaluated using a 14 camera VICON system (Oxford Metrics Ltd, UK) tracking a full body set of reflective markers (*n* = 45) at a sampling rate of 250 Hz. Model and computation details can be found in Garofolini et al. (2019). Testing speed on the treadmill was fixed for all participants at 11 km/h with the order of presentation of the conditions pseudo‐randomised, that is, combinations were balanced within each group and equal between groups. A resting period of minimum 3 min was given between conditions.

### 2.3 Data analysis

Kinematic raw data were exported to Visual 3D (C‐motion) and low‐pass filtered using a Butterworth filter (fourth order, zero lag) with a cut‐off frequency of 15 Hz. Hip, knee and ankle joint angles from the last 400 gait cycles of each condition (group‐footwear) were cut into individual cycles (foot contact (FC) to following FC) and time‐normalized to 500 samples using linear interpolation. Foot contacts were defined using the vertical component of the ground reaction force with an ascending threshold of 20N. Data were then exported to MATLAB (The MathWorks Inc., Massachusetts, US) for further analysis. The intralimb coordination was analyzed by means of hip‐knee and knee‐ankle cyclograms using only angles on the sagittal plane (i.e., flexion‐extension).

### 2.4 Cycle‐to‐cycle consistency

The cycle‐to‐cycle consistency of the cyclograms for each participant was quantified using the angular component of the coefficient of correspondence (ACC) (Field‐Fote & Tepavac, 2002), a vectorization technique that indicates the overall variability of the joint‐joint relationship for all cycles. The change in angle frame‐by‐frame is used to build a vector (*L*) with both direction and magnitude joining frame *n* to frame *n* + 1, so that

(1) $${\left|l\right|}_{n,n+1}=\sqrt{{\left(x_{n,n+1}\right)}^{2}+{\left(y_{n,n+1}\right)}^{2}}$$

where *x*_*n*,*n+1*_ and *y*_*n*,*n+1*_ represent the change in angle for the *x* joint and the *y* joint from the *n* frame to the subsequent (*n + 1*). Vectors among consecutive cycles are compared to derive the degree of dispersion of the joint‐joint values about the mean over multiple cycles for that frame pair (*a*_*n*,*n+1*_) calculated as follows:

(2) $$a_{n,n+1}=\sqrt{{\left({\bar{\cos\;\theta }}_{n,n+1}\right)}^{2}+{\left({\bar{\sin\;\theta }}_{n,n+1}\right)}^{2}}$$

where cosine (*cosθ*) and sine (*sinθ*) are derived from the *l*_*n*,*n+1*_ vector using simple trigonometry (i.e., *cosθ*_*n*,*n*+1_ = *x*_*n*,*n*+1_/*l*_*n*,*n*+1_). The average dispersion (*ā*) of all cycles is then computed as follows:

(3) $$\bar{a}=a_{1,2}+a_{2,3}+a_{3,4}\text{…}+a_{n-1,n}/n$$

where *n* is the number of cycles and *ā* is the angular component. The larger the ACC value (between 0 and 1), the less variable (less randomly distributed, more consistent) is the joint‐joint relationship. ACC values were then averaged across group and condition for further analysis.

### 2.5 Cyclogram variability

After translation of the cyclogram centroids to the origin and normalization of the angle signals to the interval [−1 1], we computed the cumulative ellipse area with half axes (*a* and *b*) corresponding to the within‐subject standard deviation of every two joint coupled angles (i.e., hip‐knee and knee‐ankle) for 20 equal bins of time‐normalized cyclograms. The sum of variance (SoV) was calculated as the cumulated elliptic area for the 20 bins as follows:

(4) $${Sum\;of\;Var}_{n}=\sum_{i=1}^{20}\pi *\;a_{n,i}b_{n,i}$$

where *n* represents the subject number and *i* is the bin number.

### 2.6 Shape difference

**FIGURE 1:**
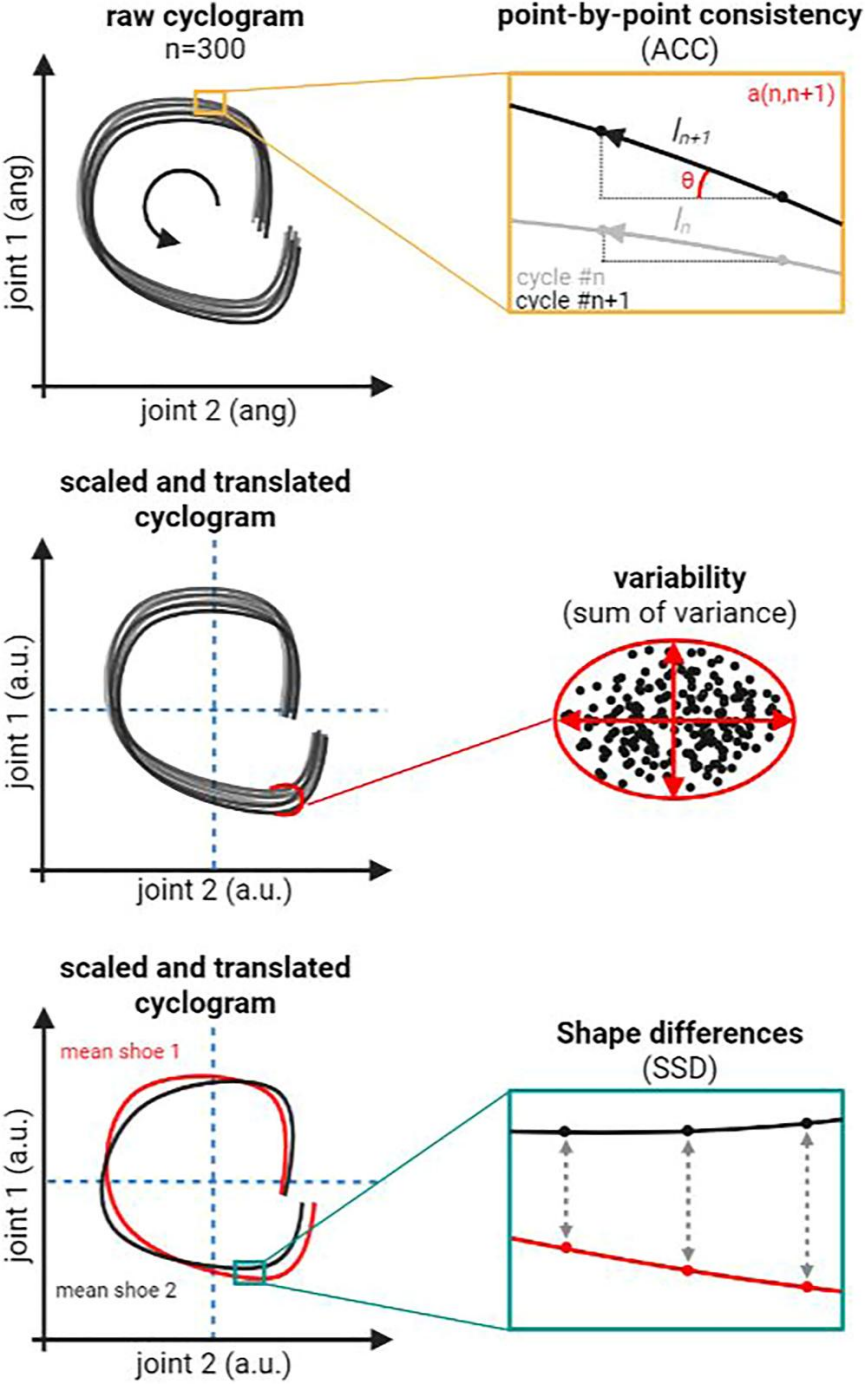
Conceptualization of the outcome variables. A cyclogram is constructed by plotting a joint angle against another coupled joint angle over a gait cycle. Consecutive cyclograms (*n* = 300) have been collected during steady state running. Three measures of intralimb coordination have been computed. The coefficient of correspondence (ACC) as a measure pf point‐by‐point and cycle‐by‐cycle consistency: a vector l is defined as the distance between two consecutive (n and n + 1) points on the cyclogram. The vectors ln and ln + 1 are compared to derive the degree of dispersion of the joint‐joint values about the mean over multiple cycles. The sum of variance is used to quantify cyclogram variability for each subject in each footwear condition. Variability is computed as the ellipse area covered by all (n = 300) cycles in 20 equally separated points along the cyclogram. The shape difference of two mean cyclograms was computed as the sum of squared distances (SSD) between the same point on the two cyclograms after translation of the centroid and rescaling of the joint angles.

The shape difference was calculated computing the average *sum of squared distances* (SSD) using the approach presented by Awai and Curt (2014). First, the mean of individuals' cyclograms was computed for each joint couple‐footwear combination, then the SSD was calculated as follows:

(5) $${SSD}_{j,k}=\sqrt{\sum_{i}{\left(\alpha_{j,i}-\alpha_{k,j}\right)}^{2}+{\left(\beta_{j,i}-\beta_{k,j}\right)}^{2}}$$

where *j* and *k* represent the two compared conditions (i.e., High vs. Low MI shoes) and *α* and *β* are the transformed and scaled joint angles at sample point *i* (Figure 1). Hence, we obtained one SSD value per individual per joint couple‐footwear combination. We used these three measures to characterize the intralimb coordination variability (Figure 1). ACC to indicate the stability of the coordinative pattern; the sum of variance to indicate the richness of the joint coupling along the coordinative pattern with higher values representing higher redundancy of the system and the SSD to measure the change in coordinative patterns between groups and within conditions (i.e., shoe type). All analysis were carried out using custom scripts in MATLAB (Math Works Inc., USA).

### 2.7 Statistical analysis

Mean and standard deviation (SD) were computed for each *Group x Shoe x Joint couple* condition. To test the hypothesis that different coordination patterns of the lower leg joint angles exists between habitual forefoot strikers and rearfoot strikers and to evaluate the influence of footwear characteristics, a mixed design 3‐factor (group x shoe x joint couple) repeated‐measures ANOVA was used to examine the interaction and main effects of between‐subject factor of foot strike pattern *Group* (2 levels: forefoot and rearfoot) and within‐subject factors of *Shoe* (3 levels: low MI, medium MI and high MI) and *Joint couple* (2 levels: hip‐knee and knee‐ankle) on the three dependent variables of variance: ACC, SSD and sum of variance. Significance was set to alpha = 0.05 for all tests. Tukey post‐hoc analysis was used to test multiple pairwise comparisons. In the absence of a standardised guideline for selecting SSD thresholds, we opted for a range of thresholds (1, 2, 3, 4 and 5 a.u.) to provide a comprehensive view of the data across different orders of magnitude. This approach allowed us to capture both subtle and more pronounced differences between subjects, thereby offering a nuanced understanding of individual variability. The selected thresholds were intended to serve as a sensitivity analysis enabling us to assess the robustness of our findings across varying levels of stringency. Paired McNemar tests were used to determine changes in the proportion of participants who displayed a change in coordinative patterns between pairs of running shoe condition comparisons (High vs. Low MI, High vs. Med MI and Low vs. Med MI). A significant McNemar *X* ^2^ (*p* < 0.05) was an indication of a difference in the proportion of runners who changed their intralimb coordinative pattern between pairs of running shoe condition comparisons. All statistics were performed using SPSS software (version 25, SPSS Inc., Chicago, IL, USA).

## 3 RESULTS

**FIGURE 2:**
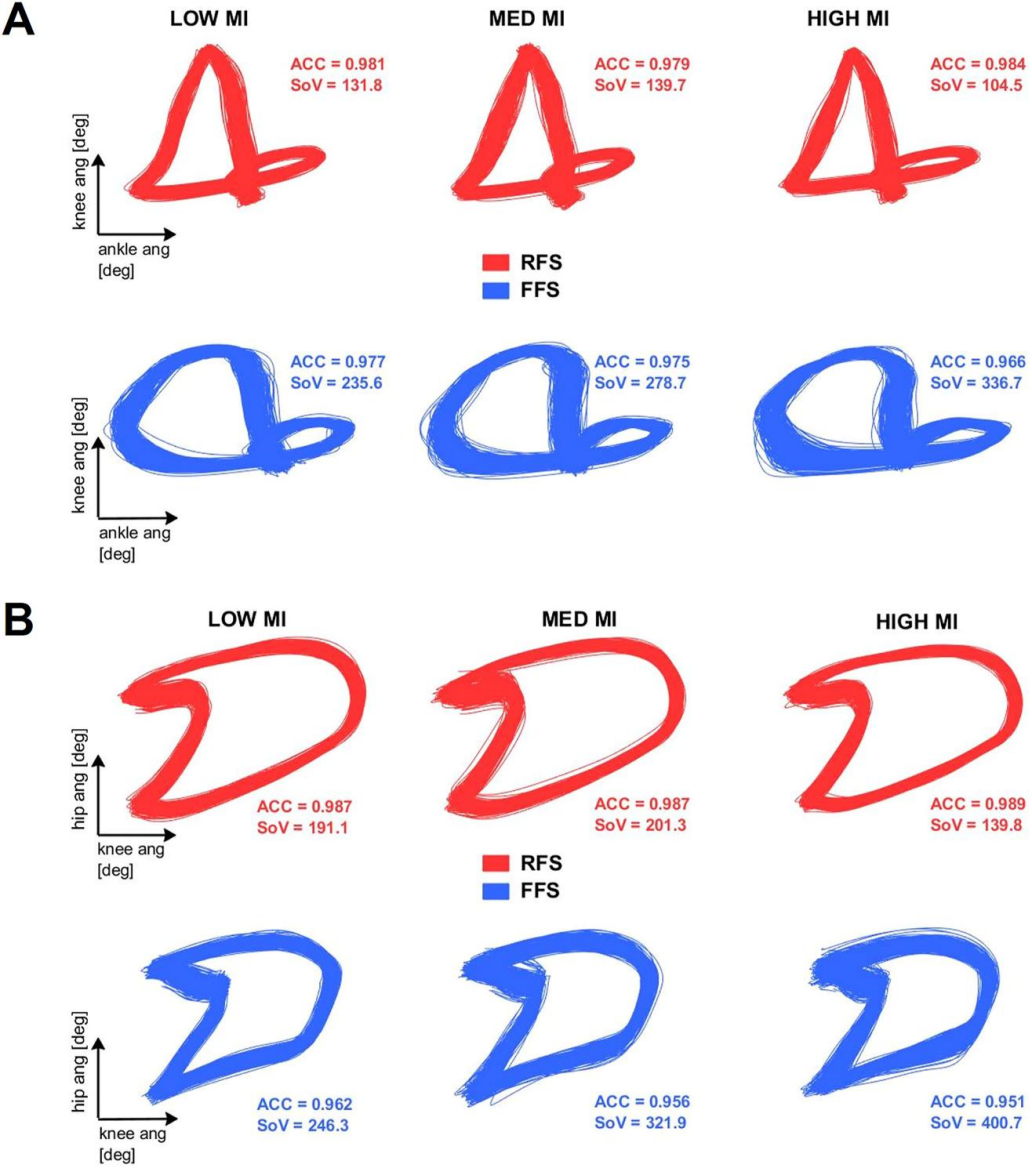
Coordination path example. (A) Ankle‐knee coordination path for a random RFS (red) and FFS (blue) subject. All 300 cycles are presented for each shoe condition: LOW MI (minimal index), MED (medium) MI, and HIGH MI. The coefficient of correspondence (ACC) and the sum of variance (SoV) values are reported for each condition. (B) Knee‐hip coordination path for a random RFS (red) and FFS (blue) subject. All 300 cycles are presented for each shoe condition: LOW MI, MED MI, and HIGH MI. The ACC and the SoV values are reported for each condition.

ANOVA assumptions for equality of variance (Levene's test) and heteroscedasticity (White's test) were met (*p* > 0.05) for the three outcome variables ACC, SoV, and SSD. Figure 2A shows ankle‐knee coordinative pattern for a FFS participant and a RFS participant in each footwear condition, while Figure 2B compares knee‐hip coordinative pattern for the same individuals.

### 3.1 Cycle‐to‐cycle consistency

**FIGURE 3:**
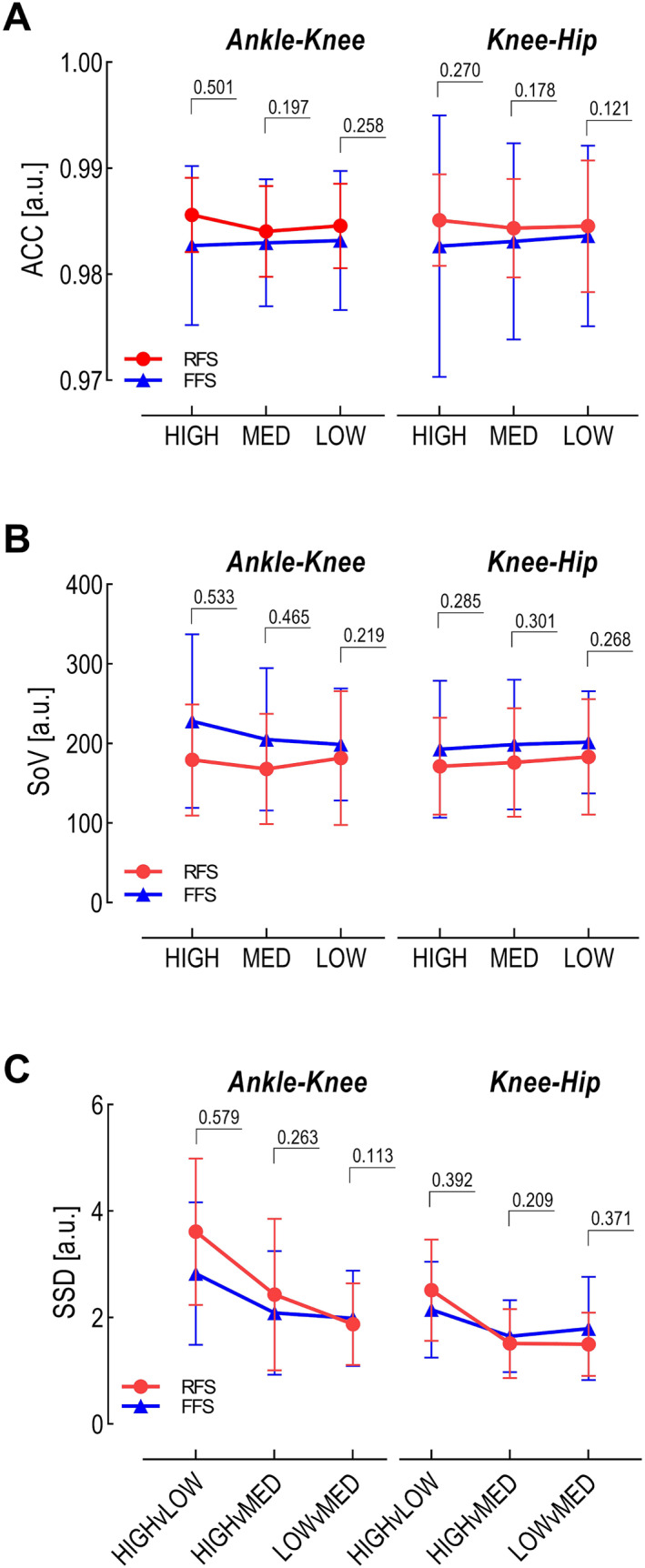
Outcome variables. Group mean with standard deviation for (A) the coefficient of correspondence (ACC) [a.u.], (B) sum of variance (SoV) [a.u.], and (C) the sum of squared distances (SSD) [a.u.]. Group comparison is made for the two joint couples: ankle‐knee, and knee‐hip, in each footwear condition: LOW MI (minimal index), MED (medium) MI, and HIGH MI. Cohen's D is used to report the effect size of the difference between groups for each Joint Couple x Shoe level. For SSD conditions are the differences between shoe types.

There were no main effects or interaction effects for ACC values indicating that cycle‐to‐cycle consistency was not statistically affected by the shoe worn (F (2, 36) = 0.1847; *p* = 0.832) and not statistically different between groups (F (1, 18) = 0.5699; *p* = 0.460; Cohen's *d* = 0.257) and joint coupling (F (1, 18) = 0.0008; *p* = 0.978). Both groups of runners have consistent cyclogram shapes at both ankle‐knee and knee‐hip level (Figure 3A). From our data, RFS generally shows higher ACC values (RFS = 0.985; FFS = 0.983), and although not significant, this difference is greater in high MI shoes as observed in Figure 3A.

### 3.2 Cyclogram variability

There were no main effects or interaction effects for the sum of variance–SoV values along the 20 equal time bins indicating that cyclogram variability was not statistically affected by the shoe worn (F (2, 36) = 0.2830; *p* = 0.755) and not statistically different between groups (F (1, 18) = 0.9388; *p* = 0.345; Cohen's *d* = 0.365) and joint coupling (F (1, 18) = 0.1663; *p* = 0.688). Both runners have similar cyclogram variability for both ankle‐knee (RFS = 176.3; FFS = 210.6) and knee‐hip coupling (RFS = 176.9; FFS = 197.6; Figure 3B). Although not statistically significant, we observed that FFS appear to exhibit greater variability in both ankle‐knee and knee‐hip coupling across all footwear conditions compared to RFS. Notably, the FFS group displayed a larger standard deviation for ankle‐knee coupling, suggesting considerable within‐group variation in movement coordination variability, in contrast to the more consistent patterns seen in the RFS group. While the greatest group difference in ankle–knee coupling is observed with high MI shoes, the groups appear most similar in terms of SoV when wearing low MI shoes (Figure 3B).

### 3.3 Shape difference

The SSD values reported in Figure 3C represent the amount of shape difference after uniform scaling and translation of the centroid. For SSD, there was a main effect of *Shoe comparison* (F (2, 36) = 26.25; *p* < 0.001) but no main effect of *Groups* (F (1, 18) = 0.6105; *p* = 0.444) or *Joint Coupling* (F (1, 18) = 4.318; *p* = 0.052). Post‐hoc analysis revealed that the shape difference between the most diverse shoes (high MI vs. low MI) is greater (*p* < 0.001) than shape difference between more similar shoes (i.e., High MI vs. Med MI, or Med MI vs. Low MI). Based on our observations (Figure 3C), the ankle–knee cyclogram shape appeared most similar between low and med MI shoes and most distinct between high and low MI shoes. We also computed the SSD for the group mean in each shoe condition for both ankle–knee and knee–hip coupling (Supplementary Figure S3). The SSD values increased for both joint couples as the shoe minimal index increased, that is, coordinative pattern shape becomes more different between groups in high MI shoes and more similar in low MI shoes.

Individual results for the shoe comparisons revealed no significant differences in proportions between the groups. As a whole, very few participants showed shape differences lower than 1 SSD in both ankle–knee (8%) and knee–hip coupling (12%) in all shoe comparisons with the majority of runners (+95%) being within 5 SSD (a.u.) (Table 1). Overall, the largest number of different movement responses was present at the ankle‐knee coupling with 11 participants showing larger differences than 3 SSD and 6 participants showing larger differences than 4 SSD. A significantly greater proportion of participants (55%) changed their ankle‐knee coordinative pattern by more than 3 SSD between the High versus Low MI conditions compared to the Low versus Med MI (10%; *X* ^2^ = 7.1; *p* = 0.008) (Table 1). Similarly, a significantly greater proportion of participants (60%) changed their knee‐hip coordinative pattern by more than 2 SSD between the High versus Low MI conditions compared to the Low versus Med MI (25%; *X* ^2^ = 4; *p* = 0.046) and compared to the High versus Med MI (20%; *X* ^2^ = 6.1; *p* = 0.013) (Table 1).

**TABLE 1:** Summary of the proportion of subjects (RFS = 10; FFS = 10 and ALL = 20) (count and percentages) with SSD [a.u.] difference in ankle‐knee and knee‐hip coupling smaller than 1, 2‐, 3‐, four‐ and five‐ between shoe comparisons.

	Ankle ‐ knee coupling														
	RFS					FFS					ALL				
	<1	<2	<3	<4	<5	<1	<2	<3	<4	<5	<1	<2	<3	<4	<5
HIGH versus LOW	0	2	**3** ^b^	5	9	0	4	6	9	9	0	6	**9** ^b^	**14** ^b^	18
%	0	20	30	50	90	0	40	60	90	90	0	30	45	70	90
HIGH versus MED	1	4	8	9	9	2	7	7	9	10	3	11	15	18	19
%	10	40	80	90	90	20	70	70	90	100	15	55	75	90	95
LOW versus MED	0	6	**10** ^b^			2	6	8	8	10	2	12	**18** ^b^	**18** ^b^	20
%	0	60	100			20	60	80	80	100	10	60	90	90	100
Mean %	3	40	70	80	93	13	57	70	87	97	8	48	70	83	95

	Knee ‐ hip coupling														
	RFS					FFS					ALL				
	**<1**	**<2**	**<3**	**<4**	**<5**	**<1**	**<2**	**<3**	**<4**	**<5**	**<1**	**< 2**	**<3**	**<4**	**<5**
HIGH versus LOW	0	**3** ^a^	7	9	10	0	5	9	9	10	0	**8** ^a^, ^b^	16	18	20
%	0	30	70	90	100	0	50	90	90	100	0	40	80	90	100
HIGH versus MED	2	**9** ^a^	10			2	7	10			4	**1** **6** ^a^	20		
%	20	90	100			20	70	100			20	80	100		
LOW versus MED	1	8	10			2	7	9	9	10	3	**15** ^b^	19	19	20
%	10	80	100			20	70	90	90	100	15	75	95	95	100
Mean %	10	67	90	97	100	13	63	93	93	100	12	65	92	95	100

^a^ Significant difference between High versus low MI and High versus med MI (*p* < 0.05).

^b^ Significant difference between High versus low MI and low versus med MI (*p* < 0.05).

A significantly greater proportion of RFS (70%) changed their ankle‐knee coordinative pattern by more than 3 SSD between the High versus Low MI conditions compared to the Low versus Med MI (0%; *X* ^2^ = 5.1; *p* = 0.023) (Table 1). Similarly, a significantly greater proportion of RFS (70%) changed their knee‐hip coordinative pattern by more than 2 SSD between the High versus Low MI conditions compared to the High versus Med MI (10%; *X* ^2^ = 4.2; *p* = 0.041).

## 4 DISCUSSION AND IMPLICATIONS

In this study, we used treadmill steady‐state running to explore the coordinative pattern and its variability within lower limb joint couplings. As the motor task is stable, we expected the coordinative pattern to represent self‐organisation of the system and its variability to represent the richness of solutions that equally solve the task.

The coordinative pattern considers coupled lower limb joints simultaneously and quantifies variability around the mean trajectory as an expression of system flexibility. Figure 2 displays 300 cycles for a representative rearfoot and forefoot striker in each shoe condition. During the stance phase, the coordinative pattern is constrained by the external forces acting on the body and from muscle activity controlling the distribution of stiffness among the joints to enable energy transfer in the limb (Zajac, Neptune, & Kautz, 2002). During swing, both the mechanical constraints inherited in the system and the external forces (i.e., gravity) define the pattern.

We hypothesized groups to have different coordinative patterns, and the difference to be more evident in high MI shoes. Statistically, this was not supported, however, we found RFS and FFS have more similar coordinative patterns in low MI shoes, while in high MI, shoes shape difference (SSD) value is the greatest for both joint couplings (Supplementary Figure S3). However, the group mean path may hide specific differences across individuals, that is, each individual within a group may have a unique coordinative pattern (Figure 2), also see Supplementary Figures S1 and S2. The comparison of the individual changes to the shoe conditions showed that the percentage of runners maintaining their coordinative pattern (<3 SSD) between the high MI and both the med MI and the low MI shoe was in the order of magnitude of about 70%–90% depending on the joint coupling. Thus, in line with the preferred movement path (Nigg, 2001), when changing shoes, the mean coordinative pattern does not change substantially (Table 1).

Coordination variability is not statistically affected by shoe type. Our findings do not reveal an effect of shoes on cycle‐to‐cycle consistency (ACC) or variability (SoV). While, rearfoot strikers tend to display greater cycle‐to‐cycle consistency in the intralimb coordinative pattern among shoe types (Figure 3A), this may be paired with less flexibility (Figure 3B). These results are in line with recent studies investigating the effect of different shoes on the preferred movement path in habitual rearfoot strikers (Weir et al., 2018). To our knowledge, our study is the first to investigate adaptation in forefoot strikers. Given that each footwear condition required a unique movement plane and therefore unique joint coupling, the strategies used by the two groups were the least different in low MI shoes. Reduced differences may be caused by the presence of cushioning materials underneath the heel or the medial aspect of the shoe in low MI shoes. By contrast, in high MI shoes, where the group shape difference is the greatest (Supplementary Figure S3), RFS may be able to ‘mimic’ the coordinative patterns of FFS by adopting a more plantarflexed ankle (Supplementary Figure S4) (McCallion et al., 2014; Squadrone et al., 2015). However, as indicated by the lower sum of variance (Figure 3B), the amount of variability available in this condition may still not be enough for the RFS to acquire an adaptable pattern. A coordinative pattern that is more variable in essence may be equipped to better respond to different shoe conditions.

From our observations, forefoot strikers tend to have greater intralimb coordination variability—SoV (Figure 3B)—partially fulfiling our first hypothesis that FFS have a larger movement solution space. Although not statistically significant, FFS tend to use more combinations of ankle‐knee coupling in all footwear conditions. Such richness of coordinative variability has been proposed to be indicative of a more flexible system (Hamill et al., 2012). The end point kinematics is mainly achieved by controlling ankle joint stiffness (Yen & Chang, 2010) and thus the relative rotation of segments. Covariance among limb segments can be reduced to two principal components that stabilize leg length and leg orientation (Ivanenko et al., 2007). Similarly here, the coordination between joint angles can be assumed to stabilize the leg length and orientation, and hence, the body center of mass position. By adapting the ankle angle, runners define the range of possible movement solutions along the other joints, so that either by compensation or collaboration, inter‐joint coupling produces stable performance.

The knee‐hip coupling was the most similar between groups (Figure 3A, Supplementary Figure S3) and the most stable (Table 1). We expected such a coupling to be the least sensitive to change, or to be the most difficult to change, based on previous studies that found the knee‐hip coupling to serve roles in both power generation and absorption during movement tasks (ElDeeb & Khodair, 2014; Wilson et al., 2016). These functional roles act as task constraints that contribute to the observed stability of knee‐hip coupling. In other words, the coupling's inherent role in efficiently generating and absorbing power makes it a stable and less variable component of the locomotor system, thereby making it less susceptible to changes even under different conditions (Argaud et al., 2019).

Indeed, our study has some limitations that warrant discussion. Utilizing a treadmill for testing may have inherently restricted the variability of gait cycles to some extent (Dingwell et al., 2001). However, the treadmill also offered the advantage of allowing us to analyze continuous gait cycles, thereby eliminating the subjective selection of cycles and enabling us to avoid the analysis of a limited number of steps. Another limitation is the lack of statistical significance when testing for differences between groups. This could be attributed to a small sample size (see Supplementary Table 2 for post‐hoc calculation of power achieved and sample size) and the unique adaptations each runner may have developed through their personal running experience (see Supplementary Figures S1 and S2). We also acknowledge that shoe weight could indeed be a contributing factor to the differences observed between the two groups. Heavier shoes could potentially alter the dynamics of the lower limb (De Wit et al., 2000; Divert et al., 2004) affecting both the range of motion and the coordination patterns of the joints involved. For instance, increased shoe weight might necessitate greater muscle activation for propulsion and stabilization (Santuz et al., 2017), thereby influencing the coordinative strategies employed by the runners (Divert et al., 2007). This could result in more pronounced differences in joint coupling, particularly in high MI shoes where the material and construction might add to the overall weight. Lastly, while our study provides valuable insights into joint coupling in the sagittal plane, we acknowledge that a comprehensive 3D analysis, that is, Trudeau et al. (2019) could offer a more nuanced understanding of these biomechanical interactions. The focus on the sagittal plane was deliberate, as the majority of the motion during running occurs in this plane (Samozino et al., 2016), making it a critical aspect of gait analysis. Despite these limitations, it is important to note that both visual inspection of the coordinative pattern and quantification of its variability serve as valuable tools for qualitatively and quantitatively describing differences between the two groups of runners. Given the small sample size, which inherently limits statistical power, a visual exploration of the data becomes particularly valuable. It allows for a more ‘rich' and nuanced understanding of the data, capturing subtleties that may not be readily apparent through quantitative metrics alone.

## 5 CONCLUSION

In summary, our study did not find significant differences in intralimb coordinative patterns between forefoot and rearfoot strikers across various shoe types partially contradicting our initial hypotheses. While shoe type did not significantly affect coordination variability, both groups showed minimal adjustments in their coordinative patterns when changing shoes. These findings suggest that individual runners have inherently stable coordinative patterns, likely influenced by genotypic traits and phenotypical adaptations, and that habitual foot strike has a minimal impact on these patterns.

## CONFLICT OF INTEREST STATEMENT

The authors report there are no competing interests to declare.

## Supporting information

Supporting Information S1
