# Supplementary material for: Quantifying coordinative patterns in steady‐state running: The impact of footwear and foot strike on joint coupling variability
Source: Eur J Sport Sci. 2024 Mar 18;24(5):566–76. doi: 10.1002/ejsc.12056 (PMC11235716; doi:10.1002/ejsc.12056)

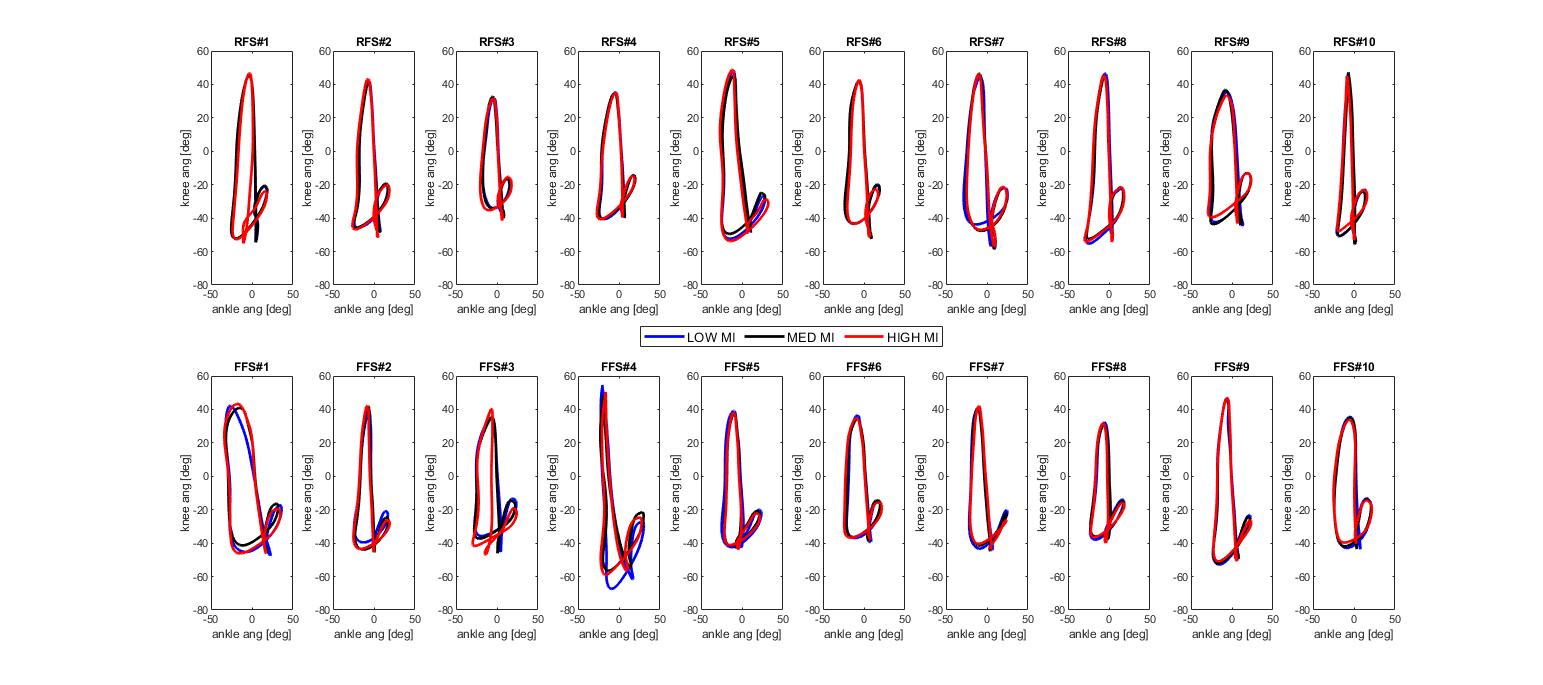
**Supplementary Figure S1.** Individual mean for ankle-knee coupling. All subjects mean for each shoe condition is presented independently. Comparison is made between high MI (minimal index), med (medium) MI, and low MI. Each individual is labelled as GROUP#ID number.


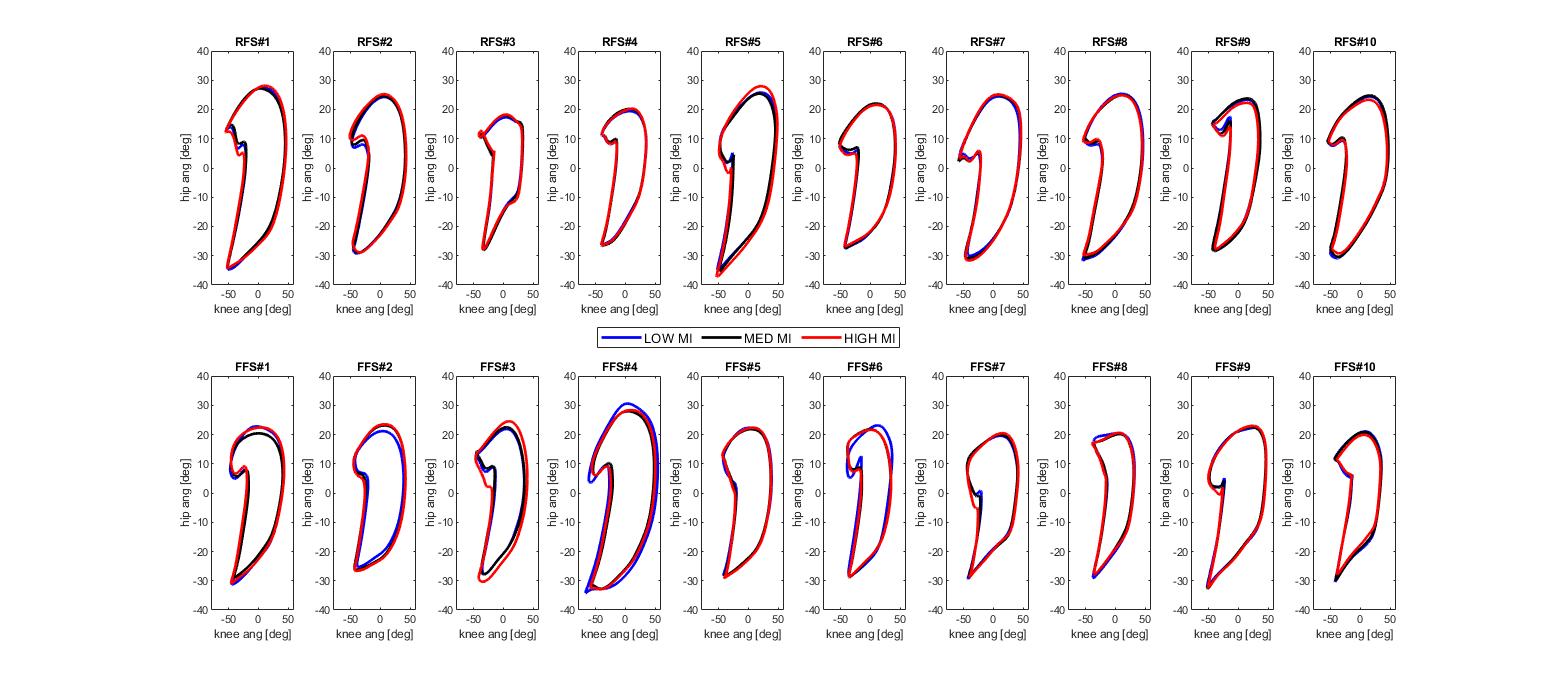
**Supplementary Figure S2.** Individual mean for knee-hip coupling. All subjects mean for each shoe condition is presented independently. Comparison is made between high MI (minimal index), med (medium) MI, and low MI. Each individual is labelled as GROUP#ID number.

**Supplementary Figure S3.** **Cyclogram shape difference**. Group mean for ankle-knee (top) and knee-hip (bottom) coordination path for each footwear condition: LOW MI (minimal index), MED (medium) MI, and HIGH MI. RFS are in red and FFS in blue. The sum of squared distances (SSD) [a.u.] is reported as a measure of shape difference between groups for shoe condition.


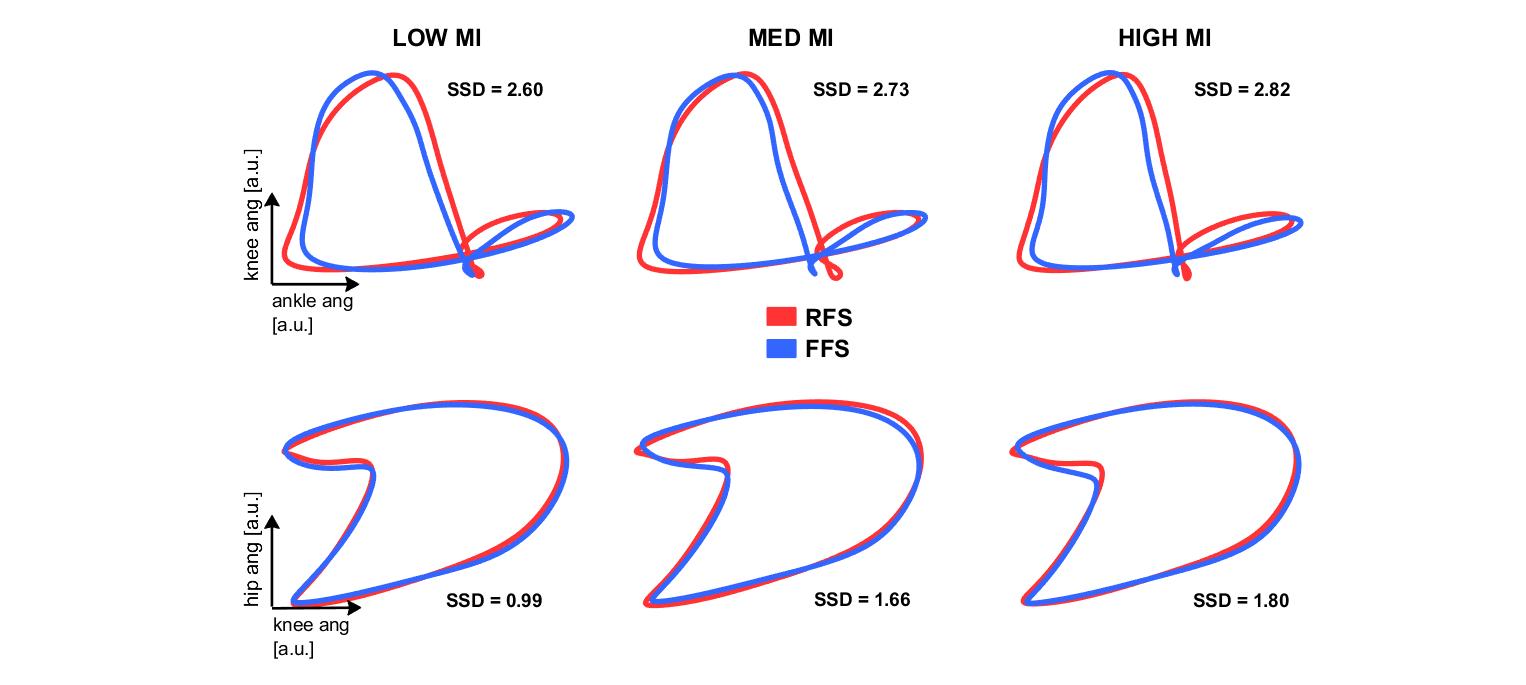


**Supplementary Figure S4.** **Raw kinematics**. Group mean hip (top), knee (middle), and ankle angle (bottom) in low, med, and high MI shoes.


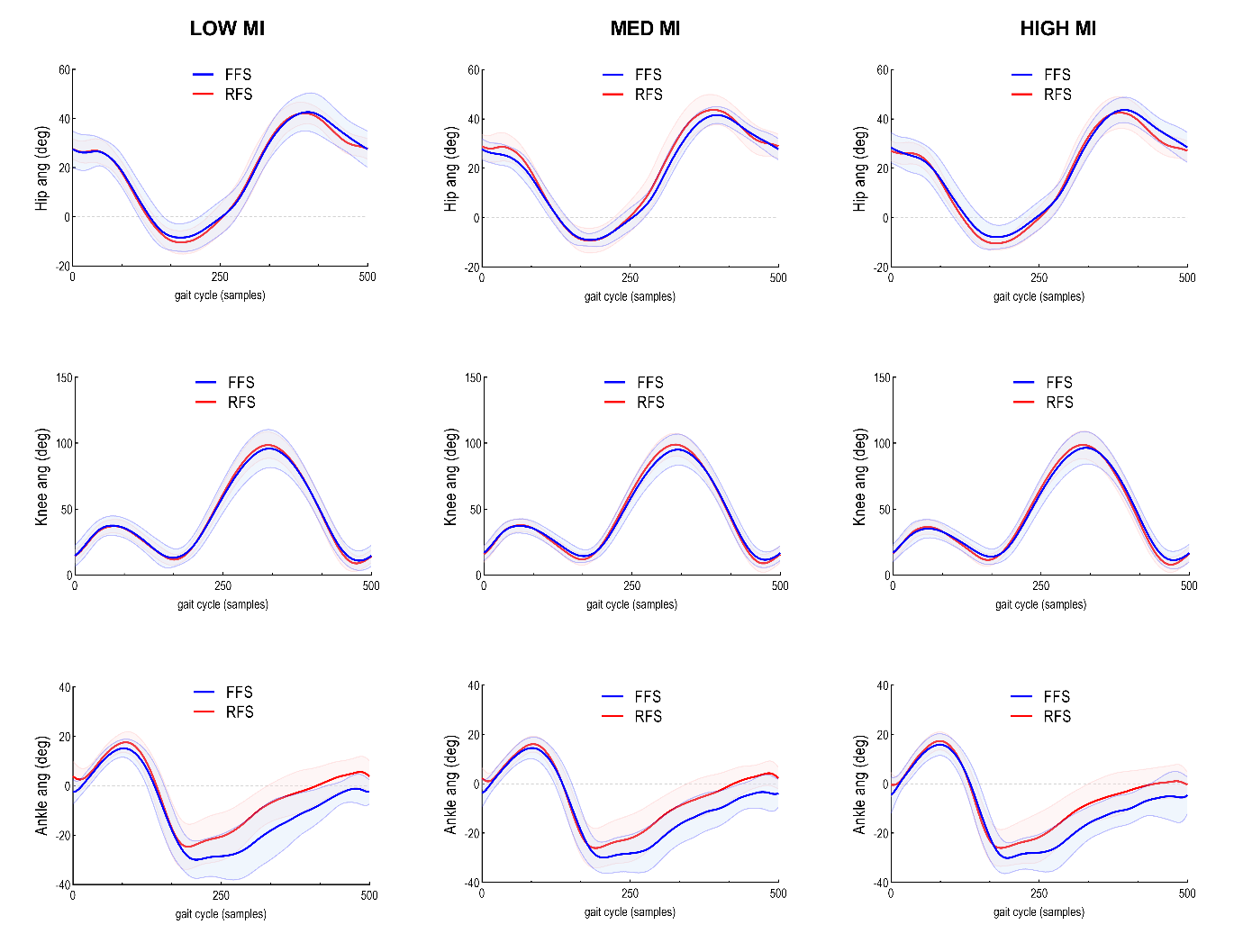


| **RFS** | **Preferred running speed (km/h)** | **Training volume (km/week)** | **Age-graded Score (%)** | **Shoe worn** |
| --- | --- | --- | --- | --- |
| Sbj 1 | 10 | 72 | 72.3 | Asics Dynaflyte |
| Sbj 2 | 11.5 | 120 | 61.7 | Asics Nimbus |
| Sbj 3 | 12 | 90 | 68.8 | New balance Vazee Pace |
| Sbj 4 | 14 | 60 | 73.4 | Nike Zoom Structure 18 |
| Sbj 5 | 12 | 50 | 60 | Asics Gel Nimbus 19 |
| Sbj 6 | 12.5 | 90 | 75 | Mizuno Wave Insipre 12 |
| Sbj 7 | 13 | 80 | 69 | Salomon S-Lab Sense 3 Ultra |
| Sbj 8 | 11 | 72 | 60.3 | Nike Epic React Flyknit |
| Sbj 9 | 11.5 | 105 | 72.4 | Asics Dynaflyte |
| Sbj 10 | 12.5 | 80 | 62.6 | Brooks beast |
| **AVG** | *12* | *82* | *67.5* |  |
| **SD** | *1.1* | *20.6* | *5.8* |  |
|  |  |  |  |  |
| **FFS** |  |  |  |  |
| Sbj 1 | 11 | 80 | 70 | Mizuno Wave Hitogami 3 |
| Sbj 2 | 13 | 90 | 61.8 | Asics Kayano 25 |
| Sbj 3 | 14 | 80 | 75.2 | Saucony Endorphin Racer 2 |
| Sbj 4 | 11 | 50 | 74.6 | Asics Kayano |
| Sbj 5 | 11 | 120 | 60.2 | Saucony libery ISO |
| Sbj 6 | 11.5 | 45 | 60.5 | Adidas Adizero |
| Sbj 7 | 12.5 | 60 | 70.7 | Nike Zoom Streak LT3 |
| Sbj 8 | 11 | 100 | 75.2 | Vibram 5 fingers |
| Sbj 9 | 12 | 105 | 61 | Saucony Freedom ISO |
| Sbj 10 | 12.5 | 105 | 77.6 | Merrell Vapor |
| **AVG** | *12* | *84* | *68.7* |  |
| **SD** | *1.0* | *25.3* | *7.1* |  |

**Supplementary Table S1.** Participants’ individual data. Preferred running speed (tested on a treadmill), training volume (self-reported), age-graded performance score, and habitual running shoe are presented. AVG = average; SD = standard deviation.

**Supplementary Table S2.** Post-hoc calculation of power achieved and sample size needed based on the computed effect sizes.


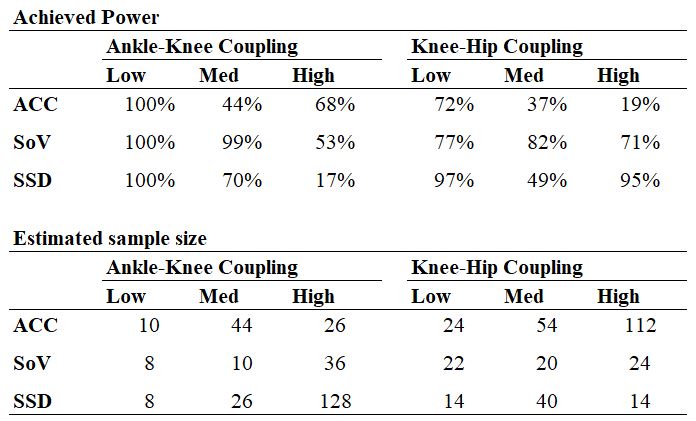

Supplement: Supplementary file 1 — Supporting Information S1 [file EJSC-24-566-s001.docx]
